# Special Issue “Regulation by Non-Coding RNAs 2025”

**DOI:** 10.3390/ijms262210885

**Published:** 2025-11-10

**Authors:** Won-Ha Lee

**Affiliations:** 1 School of Life Sciences, Kyungpook National University, Daegu 41566, Republic of Korea; whl@knu.ac.kr; 2 BK21 Plus KNU Creative BioResearch Group, Kyungpook National University, Daegu 41566, Republic of Korea; 3 Brain Science & Engineering Institute, Kyungpook National University, Daegu 41566, Republic of Korea

Non-coding RNAs (ncRNAs) have shifted from the margins of molecular biology to the core of our understanding of gene regulation, cellular plasticity, and disease pathogenesis. MicroRNAs (miRNAs), long non-coding RNAs (lncRNAs), and circular RNAs (circRNAs) interlock with chromatin, transcription, RNA processing, translation, and signal transduction, building multilayered control systems that rewire cells in development, stress, and pathology. In the past few years, three converging trends have accelerated the field: sharper mechanistic maps of lncRNA action; expanding evidence that some circRNAs can be translated by cap-independent routes; and a more sober, design-driven pipeline for ncRNA therapeutics that balances promise with lessons from early clinical programs. Together, these advances redefine ncRNAs as both biomarkers and regulators of cellular state [1,2,3].

Mechanistically, lncRNAs are now understood not as uniform “sponges,” but as diverse ribonucleoprotein scaffolds with defined subcellular localizations, modular secondary structures, and dosage-sensitive activities—often functioning at low abundance to achieve molecular specificity. This refinement underpins a burgeoning literature on lncRNA–chromatin contacts, RNA-guided recruitment of epigenetic modifiers, and modular interactions with RNA-binding proteins that sculpt transcriptional outputs. These concepts, distilled in recent comprehensive reviews, have clarified how lncRNAs act as scaffolds, decoys, guides, and enhancers across physiology and disease [1,4].

On the circRNA front, cap-independent translation has emerged from curiosity to consensus. Multiple studies now outline IRES- and m6A-dependent initiation on circRNAs, assisted by specialized factors, while computational and experimental toolkits are catching up to predict and validate translation-competent motifs at base resolution. These findings underscore a growing recognition that circRNAs can encode micro-proteins in addition to their better-known roles in sequestering miRNAs and RNA-binding proteins. This duality—scaffold and template—raises both biomedical opportunities (e.g., circRNA vaccines) and analytical challenges (e.g., distinguishing functional peptides from background translation) [3,5,6,7].

Therapeutically, miRNA, lncRNA, and circRNA programs are maturing with improved chemistry, delivery, and sequence design. The clinical path remains challenging—especially for miRNA mimics and inhibitors—but current development pipelines have become increasingly disciplined. Therapeutic indications are now selected where coordinated, network-level modulation provides a strategic advantage, and formulation design emphasizes molecular stability and immunological biocompatibility. Recent comprehensive reviews capture this evolution from exuberant discovery to a mature, engineering-driven phase of ncRNA therapeutics [2,8,9].

## 1. Highlights from the IJMS Special Issue “Regulation by Non-Coding RNAs 2025”

The nine papers collected here capture the field’s breadth—from oncology and infectious disease to insect neuroscience, hair biology, and livestock reproduction—while illustrating recurring regulatory motifs (ceRNA networks, miRNA targeting, and protein-interacting lncRNAs) and the practical genomics required to enable ncRNA discovery.

### 1.1. Cancer Therapeutics and Resistance

Mahboobnia and colleagues demonstrate that restoring the tumor-suppressive miR-142-3p can overcome tyrosine-kinase-inhibitor resistance in hepatocellular carcinoma (HCC). Mechanistically, miR-142-3p targets YES1 and TWF1, converging on YAP1 phosphorylation and autophagy pathways; functionally, miR-142-3p synergizes with lenvatinib to block growth of resistant cells. This is a quintessential ncRNA story: a single small RNA coordinates multiple nodes to reverse a complex, adaptive phenotype.

Yu et al. complement this with a network-level view, assembling HCC ceRNA maps that interlink 24 circRNAs, 28 miRNAs, and 17 hub genes across differentiation-associated modules. Experimental validation of select circuits underscores how circRNA–miRNA–mRNA triangles can surface actionable gene sets for intervention and prognosis.

### 1.2. Infectious Disease: Biomarkers and Mechanisms

Two in silico papers extend ncRNA regulation into virology and wildlife epidemiology. Salvado et al. rank miRNAs predicted to bind Parvovirus B19 RNAs (notably miR-4799-5p, miR-5690, miR-335-3p, miR-193b-5p, and miR-6771-3p), trace shared host targets, and enrich pathway hypotheses that could inform biomarker development or antiviral strategies.

Agarwal et al. analyze bat plasma miRNAs implicated in white-nose syndrome, nominating miR-543, miR-27a, miR-92b, and miR-328 as regulators at the nexus of immune response and energy metabolism. The work exemplifies how ncRNAs can integrate immunity with physiology in ecological disease contexts.

### 1.3. Neuro-Immune Regulation in Insects

Huang et al. profile a brain-expressed honeybee lncRNA (LOC113219358) that engages >100 proteins and modulates detoxification, neuronal signaling, and energy metabolism pathways; RNAi of this lncRNA reshapes enzyme activities, including AChE, GST, and CYP450. Beyond insect biology, the study is a model for proteome-level lncRNA interactomics aligned with transcriptomic shifts.

### 1.4. Skin and Hair Biology: circRNA Circuitry

Lv et al. decode a circRNA-centered circuit (circCSPP1–miR-10a–BMP7) that promotes dermal papilla cell proliferation in Hu sheep, validating ceRNA logic with luciferase assays and rescue experiments. The study joins a growing canon where circRNAs exert phenotypic control via competitive miRNA binding, here pointing to follicle growth mechanisms.

### 1.5. Genomic Infrastructure for ncRNA Discovery

Ong et al. deliver a chromosome-scale genome for the sheep-biting louse *Bovicola ovis*, scaffolding long-read sequencing with Pore-C to annotate >16,000 genes. While not an ncRNA mechanism paper per se, such high-quality genomes are instrumental for accurate ncRNA annotation, evolutionary conservation analyses, and the design of cross-species functional studies.

### 1.6. Reproductive Biology and circRNA Atlases

Liu et al. chart circRNA landscapes during goat follicular development, mapping differentially expressed circRNAs and constructing ceRNA networks around miR-324-3p, miR-202-5p, and miR-493-3p. Conservation and putative translation analyses (m6A and IRES predictions) echo the broader circRNA translation theme and offer candidates with reproductive relevance.

### 1.7. A Unifying Review on ncRNA–Protein Crosstalk

Finally, Rubina et al. summarize how miRNAs, lncRNAs, and circRNAs converge on CDH13 (T-cadherin), tuning its expression epigenetically and post-transcriptionally across cardiovascular, metabolic, oncogenic, and neurodegenerative settings. It is an elegant case study of multilayer ncRNA regulation of a single pleiotropic receptor, with clear implications for biomarker development and RNA-based therapies.

**What these papers tell us—three cross-cutting lessons**

(1) **Networks, not single nodes.** The HCC articles underline a defining feature of ncRNA interventions: efficacy often derives from coordinated modulation of a pathway ensemble (e.g., YAP1/autophagy for resistance) rather than from a single target. Analytical frameworks capable of delineating ceRNA subnetworks and multi-target miRNA repertoires will thus be pivotal for linking mechanistic insight to translational application.
(2) **Context is the mechanism.** From bat hibernation biology to honeybee neuro-immune responses and sheep hair follicle dynamics, ncRNA effects are deeply context-dependent—tethered to cell type, developmental stage, metabolic state, and environmental stressors. This resonates with broader lncRNA biology, where dosage, localization, and protein partners gate function. Designing experiments and therapies with this context sensitivity in mind is essential.
(3) **The new normal: translation-competent circRNAs.** The goat follicle atlas and the expanding review literature both assume what was once contested: many circRNAs carry features compatible with translation, and some are indeed translated. Moving forward, the integration of ribosome profiling, quantitative proteomics, and base-resolution epitranscriptomic mapping will be essential for distinguishing genuine translational events from background noise and for connecting micro-protein expression to functional phenotypes, including those relevant to vaccine design.

## 2. Outlook

The next wave of progress will likely arise from applying principles of precision engineering to the complexity of RNA biology. On the discovery front, the integration of single-cell and spatial transcriptomics with targeted RNA–protein crosslinking will sharpen causal maps of ncRNA activity in situ. On the therapeutic side, iterative design cycles encompassing chemistry optimization, delivery engineering, and rigorous on- and off-target evaluation are redefining miRNA development programs and catalyzing the emergence of lncRNA and circRNA modalities. Crucially, the field is beginning to recognize where ncRNA therapeutics are most appropriate: disease contexts in which coordinated modulation of multiple regulatory nodes confers benefit, tissues that are amenable to efficient RNA delivery, and clinical endpoints in which network-level remodeling translates into measurable therapeutic gain [2,8,9].

The contributions in this Special Issue embody that pragmatic outlook, illustrating ncRNA-mediated regulation across diverse species, tissues, and biological contexts—from overcoming therapeutic resistance to decoding infectious and ecological disease mechanisms—while highlighting the genomic resources and analytical frameworks that enable such discoveries. If the last decade taught us that most of the genome speaks in RNA, the coming one may show how to listen with enough precision to intervene. Achieving this vision will demand the same cross-disciplinary integration exemplified in this Special Issue—mechanistically grounded biology, data-driven network inference, and engineering-oriented translational design.
